# Complete Genome Sequencing of Mycobacterium heckeshornense Strain JMUB5695, Isolated from Necrotizing Granulomatous Lesions

**DOI:** 10.1128/MRA.00141-21

**Published:** 2021-07-08

**Authors:** Katsuyuki Katahira, Shinya Watanabe, Kentaro Wakamatsu, Zenzo Nagasawa, Masayuki Kawasaki, Longzhu Cui

**Affiliations:** a Department of Respiratory Medicine, National Hospital Organization Omuta National Hospital, Omuta, Fukuoka, Japan; b Division of Bacteriology, Department of Infection and Immunity, School of Medicine, Jichi Medical University, Shimotsuke, Tochigi, Japan; c Department of Medical Technology and Sciences, School of Health Sciences, Fukuoka International University of Health and Welfare, Okawa, Fukuoka, Japan; University of Maryland School of Medicine

## Abstract

We report the complete genome sequence of Mycobacterium heckeshornense strain JMUB5695, which was isolated from necrotizing granulomatous lesions in a lung cancer patient. The complete genome consists of a 4,865,109-bp chromosome with a GC content of 65.9% and contains no plasmids.

## ANNOUNCEMENT

Mycobacterium heckeshornense is a nontuberculous mycobacterium (NTM) that has been occasionally isolated from patients with pulmonary infection, tenosynovitis, lymphadenitis, lumbar spondylodiscitis, peritoneal infection, or disseminated infection (1–7). This bacterium was first characterized in 2000 (1); however, at present, only one complete genome sequence and two draft genome sequences are available, and little is known about the genetic factors underlying its pathogenesis and clinical impact. Here, we present the complete genome sequence of *M. heckeshornense* strain JMUB5695, isolated from a surgical sample of necrotizing granulomatous lesions taken from a 71-year-old male with papillary adenocarcinoma of the lung. This study was approved by the ethics committee of National Hospital Organization Omuta National Hospital (2-52).

Genomic DNA from bacterial colonies growing on Ogawa medium was extracted using a DNeasy blood and tissue kit (Qiagen, Germany). Genome sequencing and assembly were performed as described previously, with slight modifications (8), and default parameters were used except where otherwise noted. Briefly, the whole-genome sequence of *M. heckeshornense* was determined using a MinION Mk-1B device (Oxford Nanopore Technologies [ONT], UK) with a rapid sequencing kit (ONT) and a FLO-MIN106 (R9.4.1) flow cell (ONT), in addition to a MiSeq platform (Illumina, Inc. USA). A total of 89,571 reads (average size, 2,453 bp) were obtained from the ONT sequencing using the base caller Guppy v4.2.2 (ONT) and demultiplexer qcat v1.1.0 (ONT) programs. After read trimming using SeqKit v0.10.1 (9), the reads were *de novo* assembled into two contigs using Flye v2.8.1-b1676 (10) and Racon v1.4.12 (11). The contigs were polished with Pilon v1.22 (12) using short reads generated with the MiSeq platform and a Nextera XT DNA library prep kit (Illumina) and trimmed using CLC Genomics Workbench (Qiagen) (2 × 301-bp paired-end format; 2,034,378 reads; 145-fold coverage). The two existing gaps were filled by PCR amplification with KOD FX Neo (Toyobo, Japan) and primer sets (Table 1) and sequencing with a Sanger sequencer (3730xl DNA analyzer; Thermo Fisher Scientific, USA) to generate a single genome sequence. Sequencing errors and chromosome circularization were evaluated using CLC Genomics Workbench, and the resulting genome was annotated using Prokka v1.14.6 (13).

**TABLE 1:** Primers used in this study

			Nucleotide positions^*a*^	
Primer	Nucleotide sequence	Length (no. of nucleotides)	Start	End
JMUB5695_contig1-upR1	CAGGTGCAGAAGATCGACGTAGG	23	1152425	1152447
JMUB5695_contig2-upR1	ACATGGGCGTACTGAGTCAGATC	23	1164807	1164785
JMUB5695_contig1-dnF1	CCGATCAAGCATGCTCTCGTAGAG	24	1694757 (1153247)	1694734 (1153224)
JMUB5695_contig2-dnF1	GCCGTGACGTAGAAGCTCACC	21	1693797	1693817

a Relative to those in JMUB5695.

The chromosome of *M. heckeshornens*e strain JMUB5695 was 4,865,109 bp long, with a GC content of 65.9%, and it contained no plasmids. The genome sequence of JMUB5695 was closely related to those of *M. heckeshornense* strain JCM15655^T^ and Mycobacterium xenopi JCM15661^T^, the closest species to *M. heckeshornense*, with average nucleotide identities (ANIs) of 99.1% and 90.3%, respectively, as calculated using FastANI (14). The JMUB5695 genome seems to encode at least 3 type VII secretion systems and 16 ESAT-6-like homologs, which are thought to contribute to virulence (15). The genome information provided in this study will be useful as a reference and contribute to a better understanding of the pathogenicity and virulence factors of *M. heckeshornense*.

### Data availability.

The genome sequence of *M. heckeshornense* has been deposited at DDBJ/ENA/GenBank under accession no. AP024310. The BioProject accession number is PRJDB10860. The DDBJ Sequence Read Archive (DRA)/NCBI SRA accession number is DRA011225.
